# The Prognostic Impact of Lymph Node Dissection on Primary Tumor Resection for Stage IV Non–Small Cell Lung Cancer: A Population-Based Study

**DOI:** 10.3389/fonc.2022.853257

**Published:** 2022-05-05

**Authors:** Yudong Zhang, Yichi Zhang, Xinxin Cheng, Keyao Dai, Bo Xu, Shujun Liang, Minsheng Chen, Honglang Zhang, Zhenguang Chen

**Affiliations:** ^1^Department of Thoracic Surgery, The First Affiliated Hospital, Sun Yat-sen University, Guangzhou, China; ^2^Department of Cardiothoracic Surgery of East Division, The First Affiliated Hospital, Sun Yat-sen University, Guangzhou, China; ^3^Department of Thoracic Surgery and Oncology, State Key Laboratory of Respiratory Disease, National Clinical Research Center for Respiratory Disease, Guangzhou Institute of Respiratory Health, The First Affiliated Hospital of Guangzhou Medical University, Guangzhou, China; ^4^Department of Cardiothoracic Surgery, The Affiliated Hospital of Guangdong Medical University, Zhanjiang, China; ^5^Nanshan School, Guangzhou Medical University, Guangzhou, China

**Keywords:** lymph node dissection, stage IV non–small cell lung cancer, primary tumor resection, surgery, SEER database

## Abstract

**Objective:**

Selected patients with stage IV non–small cell lung cancer (NSCLC) who underwent primary tumor resection have witnessed a survival benefit. Whether additional lymph node dissection (LND) would result in a better effect remain unknown. We investigated the prognostic impact of LND on patients with stage IV NSCLC who received primary tumor resection (PTR).

**Methods:**

Patients with stage IV NSCLC who underwent PTR were identified from the Surveillance, Epidemiology, and End Results database from 2004 to 2016. Propensity-score matching was performed to minimize the confounding effect, and lung cancer-specific survival (CSS) and overall survival (OS) were compared after matching. Multivariable Cox regression was used to identify prognostic factors and to adjust for covariates in subgroup analysis. The effect of the number of lymph nodes examined on the CSS was evaluated by repeating the Cox analysis in a binary method.

**Results:**

A total of 4,114 patients with stage IV NSCLC who receive surgery met our criteria, of which 2,622 (63.73%) underwent LND and 628 patients were identified 1:1 in LND and non-LND groups after matching. Compared with the non-LND group, the LND group had a longer CSS (median: 23 vs. 16 months, p < 0.001) and OS (median: 21 vs. 15 months, p < 0.001). Multivariable regression showed that LND was independently associated with favorable CCS [hazard ratio (HR) = 0.78, 95% confidence interval (CI) 0.69–0.89, P < 0.001] and OS (HR = 0.79, 95% CI 0.70–0.89, P < 0.001). Subgroup analysis suggested that LND is an independent favorable predictor to survival in the surgical patients who were older age (>60 years old), female, T3-4, N0, and M1a stage and those who underwent sublobar resection. In addition, a statistically significant CCS benefit was associated with an increasing number of lymph nodes examined through 25 lymph nodes.

**Conclusions:**

LND with a certain range of lymph nodes number examined was associated with improved survival for patients with stage IV NSCLC who received primary tumor resection. The results may have implications for guidelines on lymph nodes management in selective advanced NSCLC for surgery.

## Introduction

Lung cancer is the leading cause of cancer-related death worldwide (1). About 85% of lung cancer pathological type was classified as non–small cell lung cancer (NSCLC), and up to 55% of which were diagnosed as stage IV due to occult onset (2). The National Comprehensive Cancer Network (NCCN) guidelines recommend surgical intervention in select cases of stage IV NSCLC with single brain or adrenal metastases but a primary tumor is otherwise T1-2, N0-1 or T3, N0 (3). Accumulating literatures suggesting that primary tumor resection (PTR) could improve survival for patients with stage IV NSCLC (4–7), particularly those with ipsilateral pleural dissemination (8–15), synchronous bone metastasis (16, 17), and extrathoracic oligometastatic (7, 18–20).

Systematic lymph node dissection (LND) or sampling during lung resection was also recommended by the NCCN guidelines for stages I–II and resectable stage IIIA NSCLC (3). From an oncological point of view, it can decrease locoregional recurrence and facilitate more accurate pathological staging for guiding subsequent therapy, which is associated with a survival benefit (21, 22). It is assumed that LND may bring better survival in patients with stage IV NSCLC who received surgery. However, little clinical evidence supports this assumption. To explore this issue, we performed a population-based study using the SEER data, to investigate the prognostic effect of LND in patients with stage IV NSCLC who received PTR, and tried to identify the surgical patients’ characteristics that were associated with survival gain from LND.

## Methods

### Patient Selection in SEER Database

The Surveillance, Epidemiology, and End Results (SEER) database accumulates massive tumor-related data and is publicly available for cancer-based epidemiology studies (23). Cases of lung cancer (C34.0-34.9) diagnosed from 2004 to 2016 were extracted from the SEER database (SEER-Stat 8.3.6) according to the site code classifications. This range was selected because the American Joint Committee on Cancer (AJCC) Tumor-Node-Metastasis (TNM) stage and Collaborative Stage (CS) information was available since 2004, and patients diagnosed after 2016 were excluded to ensure an adequate follow-up time. Adult patients were included by following criteria: 1) pathologically confirmed NSCLC (the major histologic subtypes of adenocarcinoma and squamous cell carcinoma); 2) diagnosed as stage IV (the TNM stage was reclassified to the AJCC eighth edition based on the accessible information); and 3) diagnosed as the first primary malignancy in lifetime; 4) with one primary site and received the primary tumor resection. Patients were excluded if: 1) information on primary tumor position, TNM stage, surgical status, regional nodes examined, survival month, or treatment modality was unavailable; 2) the patients had T0 local disease.

### Statistical Analysis

The patients were divided into LND and non-LND groups according to with or without LND during surgery (a binary variable). The LND indicates that at least one regional lymph node (LN) was examined without distinguishing between systemic mediastinal or lobe-specific LND, whereas the non-LND means no node examined. Propensity-score matching (PSM) was used to balance baseline covariates. A logistic regression model was built to calculate the propensity scores of the following covariates: age, gender, histology, differentiation, tumor position, TNM stage, metastatic site resection, and chemoradiotherapy. The caliper was set at 0.01. The LND group was matched with the non-LND group by 1:1 using the nearest propensity score without replacement. Covariates were considered comparable when standardized mean differences (SMDs) were below 0.10.

Cancer-specific survival (CSS) was defined as the date of diagnosis to the date of cancer-specific death, and overall survival (OS) was the time from diagnosis to death from any cause; both were estimated by the Kaplan–Meier method and compared with the log-rank test between two groups. Multivariable Cox proportional hazard regression model was constructed to identify factors associated with CCS and OS and was applied to adjust covariates in the subgroup analysis for exploring whether LND would associate with survival benefit in the particular population. Multivariable regression included all variables with p < 0.15 in the univariable analysis.

The impact of the number of LNs examined was evaluated in a binary way by repeating the Cox proportional hazards regression model for variable adjustment as follows. Survival was incrementally compared between any patient with 0 to a certain number of LNs examined and those with greater than that specific number. The base reference was the whole cohort of non-LND. This accumulative method was explained (24). Hypothesis testing was conducted in two-sided with R software (version 3.6.1, https://cran.r-project.org/). A p-value of 0.05 was used to define significance and was presented without adjustment for multiplicity.

## Results

### Baseline Characteristics

**Figure 1:**
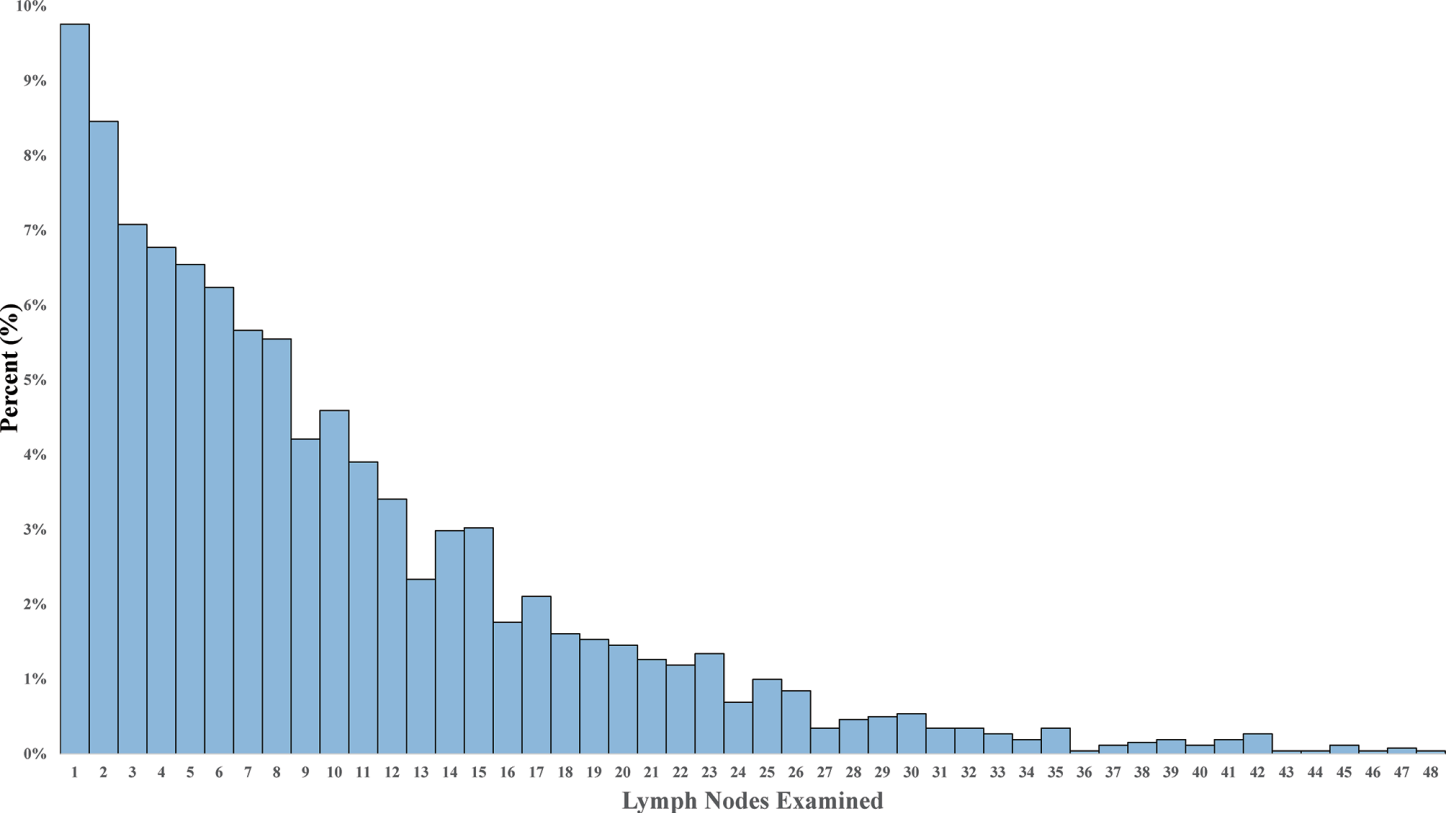
The distribution of lymph nodes examined in stage IV non–small cell lung cancer patients with lymph node dissection.

A design flow chart was shown (**Supplementary Figure 1**). Of all 4,114 eligible patients who underwent PTR, 2,622 (63.73%) were LND group. The distribution of LNs examined was shown [median: 7; interquartile range (IQR), 3 to 13] (**Figure 1**). In addition, 1,492 (36.27%) were non-LND groups. Distinctive differences in age, histology, differentiation, tumor position, TNM stage, surgical types, chemoradiotherapy, and metastasis site resection were noted between the two groups. In particular, LND was associated with a lower T/N descriptor, which indicated that the baseline characteristics of the two groups were not comparable. After the 1:1 PSM, 1,256 patients with stage IV NSCLC underwent with or without LND in surgery were enrolled in the survival analysis. Baseline characteristics showed balance (**Table 1**).

**Table 1:** Baseline characteristics for patients with Stage IV NSCLC before and after PSM.

	Before PSM		SMD	After PSM		SMD
	Non-LN dissection	LN dissection		Non-LN dissection	LN dissection	
	(n = 1,492)	(n = 2,622)		(n = 628)	(n = 628)	
**Age**						
<60	470 (31.5)	909 (34.7)	0.376	194 (30.9)	215 (34.2)	<0.001
60–75	738 (49.5)	1,333 (50.8)		312 (49.7)	312 (49.7)	
>75	284 (19.0)	380 (14.5)		122 (19.4)	101 (16.1)	
**Gender**						
Male	746 (50.0)	1,297 (49.5)	0.742	325 (51.8)	305 (48.6)	0.032
Female	746 (50.0)	1,325 (50.5)		303 (48.2)	323 (51.4)	
**Histology**						
Squamous carcinoma	182 (12.2)	456 (17.4)	0.053	91 (14.5)	79 (12.6)	<0.001
Adenocarcinoma	1,096 (73.5)	1,695 (64.6)		444 (70.7)	428 (68.2)	
Other	214 (14.3)	471 (18.0)		93 (14.8)	121 (19.3)	
**Differentiation**						
Well	168 (11.3)	203 (7.7)	<0.001	68 (10.8)	69 (11.0)	<0.001
Moderately	425 (28.5)	834 (31.8)		170 (27.1)	149 (23.7)	
Poorly	464 (31.3)	1,183 (45.1)		227 (36.1)	252 (40.1)	
Undifferentiated	46 (3.1)	104 (4.0)		25 (4.0)	27 (4.3)	
Unknown	389 (26.1)	298 (11.4)		138 (22.0)	131 (20.9)	
**Position**						
Peribronchial	12 (0.8)	27 (1.0)	0.030	6 (1.0)	7 (1.1)	0.010
Intralobar	1,217 (81.6)	2,342 (89.3)		521 (83.0)	539 (85.8)	
Both	28 (1.9)	85 (3.2)		12 (1.9)	12 (1.9)	
Unknown	235 (15.8)	168 (6.4)		89 (14.2)	70 (11.1)	
**AJCC T status**						
T1	237 (15.9)	468 (17.8)	0.183	117 (18.6)	149 (23.7)	0.051
T2	290 (19.4)	1,069 (40.8)		161 (25.6)	129 (20.5)	
T3	281 (18.8)	446 (17.0)		108 (17.2)	114 (18.2)	
T4	684 (45.8)	639 (24.4)		242 (38.5)	236 (37.6)	
**AJCC N status**						
N0	822 (55.1)	1,264 (48.2)	0.297	342 (54.5)	329 (52.4)	<0.001
N1	109 (7.3)	519 (19.8)		64 (10.2)	66 (10.5)	
N2	431 (28.9)	770 (29.4)		183 (29.1)	202 (32.2)	
N3	130 (8.7)	69 (2.6)		39 (6.2)	31 (4.9)	
**AJCC M status**						
M1a	420 (28.2)	485 (18.5)	0.376	161 (25.6)	144 (22.9)	<0.001
M1b	348 (23.3)	679 (25.9)		139 (22.1)	131 (20.9)	
M1	724 (48.5)	1,458 (55.6)		328 (52.2)	353 (56.2)	
**Primary surgery**						
Sublobar resection	1,243 (83.3)	523 (19.9)	0.355	430 (68.5)	416 (66.2)	0.060
Lobectomy	231 (15.5)	1,814 (69.2)		180 (28.7)	191 (30.4)	
Pneumonectomy	18 (1.2)	285 (10.9)		18 (2.9)	21 (3.3)	
**Radiation**						
No	1,039 (69.6)	1,569 (59.8)	0.221	418 (66.6)	399 (63.5)	<0.001
Yes	453 (30.4)	1,053 (40.2)		210 (33.4)	229 (36.5)	
**Chemotherapy**						
No/Unknown	590 (39.5)	1,150 (43.9)	0.110	268 (42.7)	279 (44.4)	0.010
Yes	902 (60.5)	1,472 (56.1)		360 (57.3)	349 (55.6)	
**Surgery to metastasis site**						
No	1,085 (72.7)	1,576 (52.8)	0.174	379 (60.4)	371 (59.1)	<0.001
Yes	407 (27.3)	1406 (47.2)		249 (39.6)	257 (40.9)	

PSM, propensity score matching; NSCLC, non–small cell lung cancer; LN, lymph node; AJCC, American Joint Committee on Cancer.

### Impact of Lymph Node Dissection on Survival

**Figure 2:**
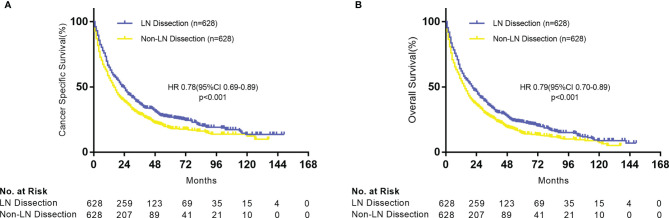
Kaplan–Meier plot of survival outcomes for patients with stage IV NSCLC according to lymph node dissection. **(A)** Cancer-specific survival. **(B)** Overall survival.

In the matched cohort of stage IV NSCLC surgical patients, the LND group had significantly longer CSS and OS. The median CSS time of 23 months (IQR, 7–38) for the LND group versus 16 months (IQR, 5–31) for the non-LN resection group [HR = 0.78, 95% confidence interval (CI) 0.69–0.89, P < 0.001] (**Figure 2A**). The median OS was 21 months (IQR, 7–37) and 15 months (IQR, 5–31) in LND and non-LND groups, respectively (HR = 0.79, 95% CI 0.70–0.89, P < 0.001) (**Figure 2B**).

### Lymph Node Dissection as an Independent Prognostic Factor

**Figure 3:**
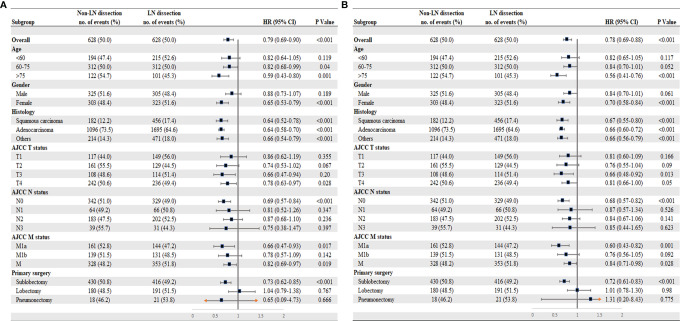
Subgroup analysis for patients with stage IV NSCLC according to lymph node dissection. **(A)** Cancer-specific survival. **(B)** Overall survival. *M1a (separate tumor nodule in a contralateral lobe, or malignant pleural effusion); M1b (single or multiple extrathoracic metastases); M1 (either M1a or M1b).

In the multivariable Cox analysis of the matched cohorts, LND was independently associated with improved CSS (HR = 0.78, 95% CI 0.69–0.90, P < 0.001) and OS (HR = 0.78, 95% CI 0.69–0.88, P < 0.001) in stage IV NSCLC surgical patients. Age, gender, TNM stage, differentiation, surgery types, chemoradiotherapy, and metastatic sites resection were also independent prognostic factors (**Table 2**). We further explored whether LND was associated with survival benefits in specific subgroups of the surgical population. As a whole, both CSS (**Figure 3A**) and OS (**Figure 3B**) showed similar results in corresponding subgroups. The potential favorable features for the surgical patients who received LND included: old (>60 years old), females, T3-4, N0, M1a, and sublobar resection (SLR).

**Table 2:** Multivariable analysis for lung cancer–specific survival in patients with NSCLC with surgery.

Characteristic	Cancer-Specific Survival		Overall Survival	
	HR (95% CI)	P Value	HR (95% CI)	P Value
**Age**				
<60	1.00 (reference)		1.00 (reference)	
60-75	1.22 (1.05–1.43)	0.012	1.23 (1.06–1.43)	0.007
>75	1.89 (1.55–2.31)	<0.001	1.81 (1.49–2.20)	<0.001
**Gender**				
Male	1.00 (reference)		1.00 (reference)	
Female	0.69 (0.60–0.79)	<0.001	0.70 (0.62–0.80)	<0.001
**Histology**				
Squamous carcinoma	/	/	1.00 (reference)	
Adenocarcinoma	/	/	0.75 (0.62–0.90)	0.002
Other	/	/	0.97 (0.77–1.20)	0.754
**Differentiation**				
Well	1.00 (reference)		/	/
Moderately	1.52 (1.16–2.00)	0.002	/	/
Poorly	1.61 (1.24–2.09)	<0.001	/	/
Undifferentiated	1.73 (1.17–2.56)	0.006	/	/
Unknown	1.55 (1.17–2.05)	0.002	/	/
**Position**				
Peribronchial	1.00 (reference)		1.00 (reference)	
Intralobar	1.31 (0.67–2.56)	0.437	1.43 (0.76–2.71)	0.27
Both	2.54 (1.13–5.71)	0.024	2.68 (1.24–5.80)	0.012
Unknown	1.33 (0.66–2.67)	0.419	1.44 (0.75–2.79)	0.278
**AJCC T status**				
T1	1.00 (reference)		1.00 (reference)	
T2	1.16 (0.95–1.43)	0.154	1.09 (0.90–1.32)	0.368
T3	1.26 (1.00–1.60)	0.054	1.29 (1.04–1.61)	0.022
T4	1.34 (1.10–1.62)	0.003	1.35 (1.13–1.61)	0.001
**AJCC N status**				
N0	1.00 (reference)		1.00 (reference)	
N1	1.60 (1.25–1.95)	<0.001	1.48 (1.19–1.83)	<0.001
N2	1.51 (1.30–1.76)	<0.001	1.53 (1.32–1.78)	<0.001
N3	1.72 (1.28–2.29)	<0.001	1.78 (1.34–2.36)	<0.001
**AJCC M status**				
M1a	1.00 (reference)		1.00 (reference)	
M1b	1.34 (1.07–1.68)	0.01	1.42 (1.15–1.76)	0.001
M1	1.31 (1.09–1.59)	0.005	1.35 (1.12–1.62)	0.001
**Primary surgery**				
Sublobectomy	1.00 (reference)		1.00 (reference)	
Lobectomy	0.70 (0.60–0.82)	<0.001	0.69 (0.60–0.80)	<0.001
Pneumonectomy	0.49 (0.33–0.74)	0.001	0.44 (0.29–0.64)	<0.001
**Lymph node dissection**				
No	1.00 (reference)		1.00 (reference)	
Yes	0.78 (0.69–0.90)	<0.001	0.78 (0.69–0.88)	<0.001
**Radiation**				
No	1.00 (reference)		1.00 (reference)	
Yes	1.44 (1.24–1.67)	<0.001	1.50 (1.30–1.73)	<0.001
**Chemotherapy**				
No	/	/	1.00 (reference)	
Yes	/	/	0.74 (0.64–0.84)	<0.001
**Surgery to metastasis site**				
No	1.00 (reference)		1.00 (reference)	
Yes	0.77 (0.69–0.84)	<0.001	0.76 (0.70–0.83)	<0.001

PSM, propensity score matching; NSCLC, non–small cell lung cancer; LN, lymph node; AJCC, American Joint Committee on Cancer.

### Prognosis of Each Additional Lymph Node Examination

**Figure 4:**
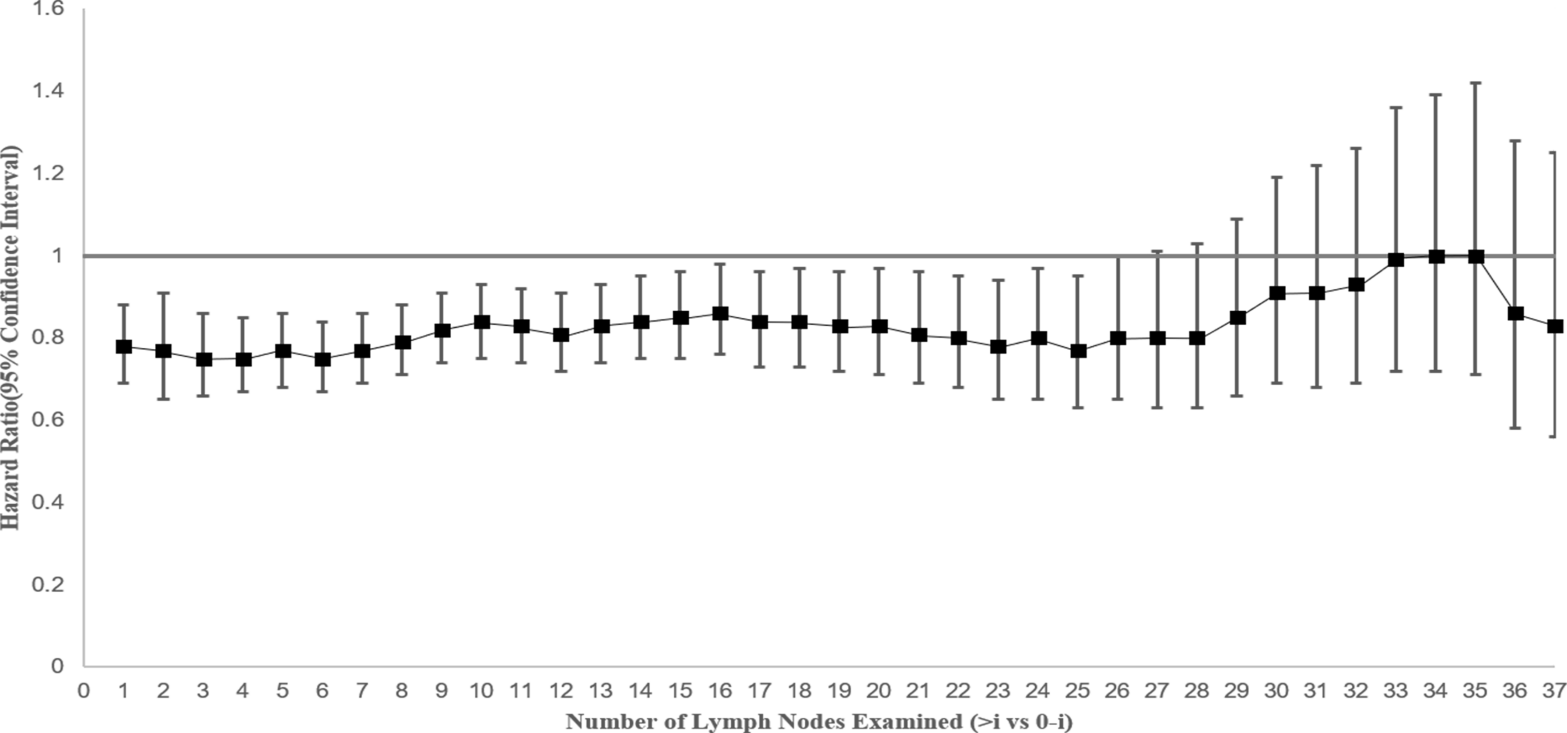
Hazard for cancer-specific survival and 95% confidence interval by extent of lymph node evaluation by comparing greater than “i” versus “0 to i” lymph nodes (where “i” is the number of lymph nodes examined) among patients with stage IV NSCLC.

Cox proportional hazards regression model was performed to determine the adjusted mortality benefit of examining every additional LN. A statistically significant CSS benefit was associated with each additional LN examined through 25 LNs, which suggests that higher volume of examined LNs in stage IV NSCLC surgical patients might improve survival (**Figure 4**).

## Discussion

It is now virtually universally accepted that there are subsets of patients with stage IV NSCLC who benefit from curative intent therapy, with surgery being done very selectively (5, 25). Studies suggested that PTR was associated with improved survival in patients with NSCLC with pleural carcinomatosis or extrathoracic metastatic, particularly for those with single-organ metastasis (7, 11, 14, 17, 19, 20). Further studies identified the specific group with <60 years old, female, adenocarcinoma, well differentiation, tumor site in lobe, T1-2, N0, and M1a that were potentially associated with more favorable survival (7, 26).

Systematic LND and sampling remains the standard part in surgical treatment to early and middle stage NSCLC, for that it could reduce local recurrence and guide subsequent therapy by determining the pathological stage, which could result in improved survival (27–29). For some reasons, few reports are concerned with the oncological benefit of LND in patients with advanced NSCLC with surgery. In clinical practice, the conduct of LND will be hindered by intraoperative adverse conditions such as LN adhesion, tissue edema, and complex anatomy for avoiding unexpected bleeding. Another more important reason is common sense holds that LND should not be a routine procedure in metastatic solid tumor patients.

### The Lymph Nodes Dissection Effect

Whether LND would bring better outcomes in patients with advanced NSCLC who receive surgical treatment remains unknown. In this study, the clinical significance of LND in patients with stage IV NSCLC who underwent PTR was investigated using data from the SEER database. The results show that LND was an independent prognostic factor associated with improved survival, especially in the surgical patients who were older age (>60 years old), female, T3-4, N0, and M1a and those treated with SLR. In addition, a CCS benefit was associated with an increasing number of LNs examined through 25 LNs.

The possible reasons for LND that may have a survival benefit in stage IV NSCLC are as follows: 1.) LND eliminates tumor cells in draining LN regions as they could tip the balance against anti-tumor immune response and facilitate the spread of metastatic tumor cells (30); 2.) pathological LN metastasis with lymphatic invasion does present in clinically node-negative (N0), which is associated with increased rates of distant and LNs recurrence (31), and lymphadenectomy could stop latent self-seeding of primary tumor cells through lymphatic stations by clearing potential positive LNs; 3.) salvage surgery after targeted therapy could contribute to prolonged OS by reducing the local tumor burden (32), and LND may play a similar role in lymphatic nodes involved.

### Lymph Nodes Dissection and Surgery

The LND was performed on the basis of surgery; therefore, the relation between LND and surgery is worth discussing. The previous studies showed that surgery was associated with more survival benefits in patients with stage IV NSCLC with lower T stage as T1-2 (26) compared with those with higher T, and lobectomy might have better survival versus SLR (33). This present study suggests LND might benefit stage IV NSCLC patients who underwent surgery with higher T stage like T3-4 and those who received SLR, as compared with the non-LND. We speculate that LND is complementary to surgery in survival, because the more advanced T stage (the larger tumor size) and the less excision (like SLR) would lead to the greater probability of occult lymphatic metastasis, which is associated with worse survival due to regional recurrence. Thus, the LND may bring survival benefit by the possible mechanism mentioned above. We suggest that when limited resection was applied to the larger primary tumors in patients with stage IV NSCLC for palliative or curative intent, the significance of LND should be more emphasized. This assumption is consistent with the finding in early-stage NSCLC. Studies revealed that greater extent of the LND should be done to larger primary tumor size during surgery in clinical stage I NSCLC regarding survival (24) and indicated that SLR with a more extensive lymphadenectomy was associated with equivalent survival with lobectomy in stage I tumors < 2 cm (34).

### The Volume of Lymph Nodes Examined

Whether higher numbers of examined LNs in patients with stage IV NSCLC would improve survival is also worth discussing. A minimum of 10 examined LNs for dissection or sampling for T(1-3)N(0)M(0) NSCLC patients was recommended for better prognosis (35). The previous study found that a greater number of LNs examined are associated with more accurate node staging and better long-term survival in resected early-stage NSCLC and recommended the 16 LNs as the cutoff point for evaluating the quality of LN examination (36). In this study, we found similar results in stage IV NSCLC and suggested that the increasing number of LNs examined in a range of 1 to 25 nodes was associated with survival benefits. These findings show the efficacy of LNs numbers management not only in the early-stage NSCLC but also in the advanced NSCLC.

### Limitations

Although this study supports the clinical efficacy of LND in stage IV NSCLC surgery patients, the results should be interpreted with caution for several limitations. 1.) It is not clear what criteria were used for selecting patients with stage IV NSCLC for surgery with or without LND in the database, which was influenced by surgeons’ personal dispositions. These unavailable data such as physical performance status and preoperative comorbidities serve as an uncontrollable confounding factor and those assessable baseline variables before matching were unbalanced. These indicate the high heterogeneity of the study population. Although we used the PSM to enhance the comparability between the two groups and performed multivariable Cox regression to adjust covariables for validation, what we could draw in this study was merely the association but not the causality. 2.) Metastatic information from the SEER database was incomplete. Only the metastatic site distribution could be roughly collected, and the data on metastatic lesions number were unavailable. Information on the locations of retrieved LNs is also unknowable from this dataset. In addition, the transformation of the AJCC TNM stage across different editions was not entirely complete due to some incompatible. 3.) Systematic therapy data on chemotherapy timing and types, metastatic sites radiation, and targeted therapy and immunotherapy were not available in the database, which is another limitation, because they are currently prevalent in the treatment for stage IV NSCLC and may influence the practical meaning of surgery with LND. 4.) As the study factor, LND is indicated as a binary variable; moreover, as the term, it covers the varying meaning of quality of pathologic nodal evaluation (removal, sample, or examine). In addition, the number of LNs dissected may associate with different centers’ experience and the way of removing and counting LNs (LNs fragments or complete LNs) may not be standardized across institutions. Therefore, we can only determine the number of LNs examined, which is not necessarily the true number of LNs resected.

Ideally, prospective trials should be warranted to validate the findings. Nevertheless, a prospective study may not be practically feasible to conduct in a single-center due to limited cases of patients with stage IV NSCLC with surgery. The SEER database provided real-world data that may facilitate understanding the impact of LND in surgery on advanced lung cancer. Considering the absence of high-level evidence, the population-based study with the SEER database is believed to be the most ideal approach to investigate this issue.

## Conclusion

This study shows that LND with a certain range of lymph nodes number examined is associated with improved survival of patients with stage IV NSCLC who receive primary tumor resection, particularly in those who were older (>60 years old), female, T3-4, N0, and M1a stage and those underwent SLR, as compared with non-LND. We suggest that when surgery was indicated in patients with stage IV NSCLC, the significance of LND should be emphasized. The results may have implications for guidelines on lymph nodes management in selective advanced NSCLC for surgery.

## Data Availability Statement

The original contributions presented in the study are included in the article/**Supplementary Material**. Further inquiries can be directed to the corresponding author.

## Author Contributions

YDZ and ZC contributed to the study design. YCZ and YDZ contributed to data analysis and interpretation. YCZ, YDZ, KD, XC, and BX contributed to data collection. All authors contributed to the drafting of the article and to its revisions. All authors contributed to the article and approved the submitted version.

## Conflict of Interest

The authors declare that the research was conducted in the absence of any commercial or financial relationships that could be construed as a potential conflict of interest.

## Publisher’s Note

All claims expressed in this article are solely those of the authors and do not necessarily represent those of their affiliated organizations, or those of the publisher, the editors and the reviewers. Any product that may be evaluated in this article, or claim that may be made by its manufacturer, is not guaranteed or endorsed by the publisher.

## Abbreviations

SEER, Surveillance, Epidemiology, and End Results; NSCLC, non–small cell lung cancer; PTR, primary tumor resection; LND, lymph node dissection; LNs, lymph nodes; CSS, cancer-specific survival; OS, overall survival; SLR, sublobar resection; PSM, propensity-score matching; IQR, interquartile range.
