# Supplementary figures and images for: The Prognostic Impact of Lymph Node Dissection on Primary Tumor Resection for Stage IV Non–Small Cell Lung Cancer: A Population-Based Study
Source: Front Oncol. 2022 May 5;12:853257. doi: 10.3389/fonc.2022.853257 (PMC9117632; doi:10.3389/fonc.2022.853257)

Supplementary Figure 1

Design flow chart

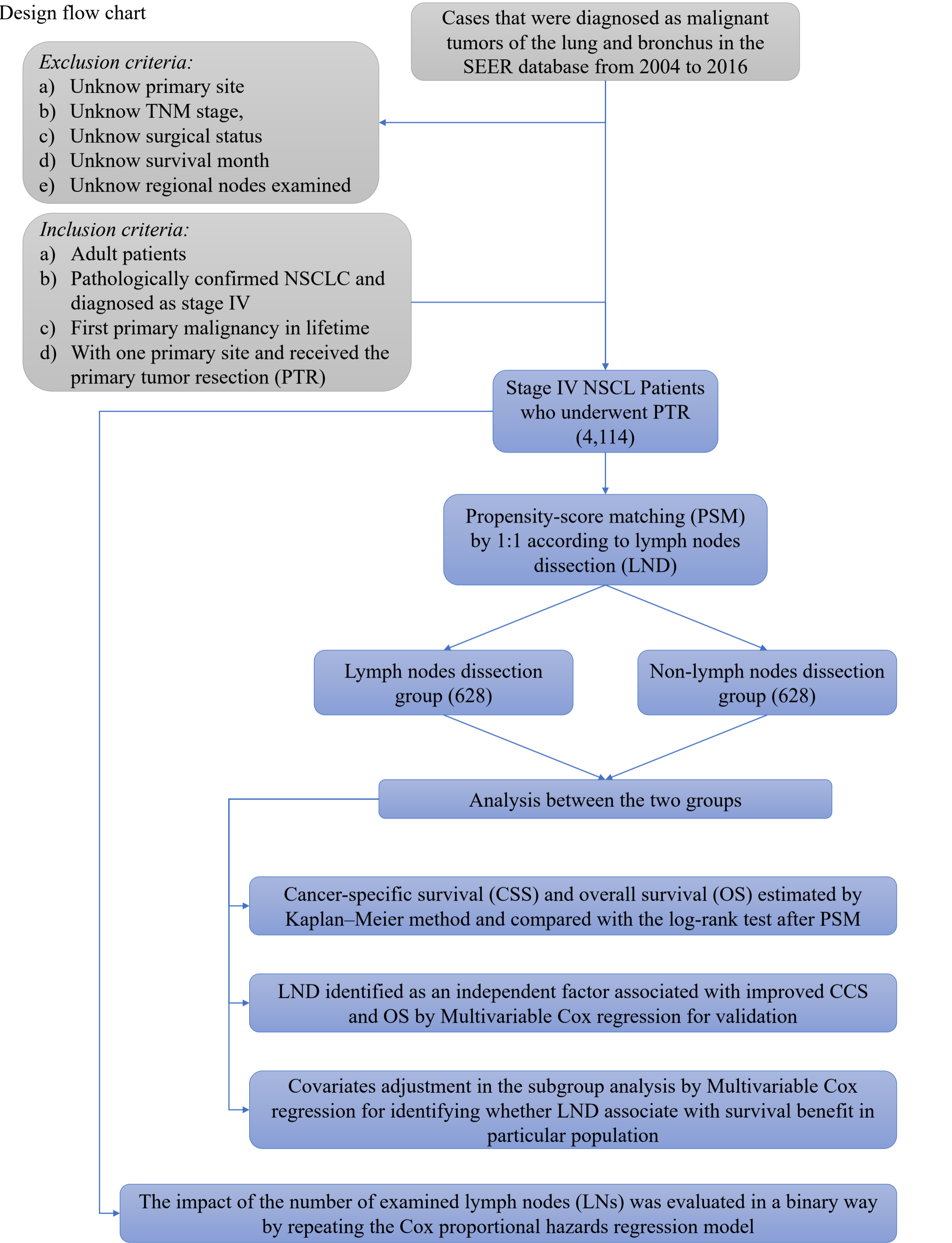

Supplement: Supplementary file 1 [file Presentation_1.pdf]
